# An Oligomeric Sulfated Hyaluronan and Silk-Elastinlike Polymer Combination Protects against Murine Radiation Induced Proctitis

**DOI:** 10.3390/pharmaceutics14010175

**Published:** 2022-01-12

**Authors:** Douglas Steinhauff, Mark Martin Jensen, Ethan Griswold, Jolanta Jedrzkiewicz, Joseph Cappello, Siam Oottamasathien, Hamidreza Ghandehari

**Affiliations:** 1 Department of Biomedical Engineering, University of Utah, Salt Lake City, UT 84112, USA; douglas.steinhauff@utah.edu (D.S.); Ethan.Griswold@utah.edu (E.G.); 2 Utah Center for Nanomedicine, University of Utah, Salt Lake City, UT 84112, USA; 3 Department of Surgery, Massachusetts General Hospital, Harvard Medical School, Boston, MA 02114, USA; MMJENSEN@mgh.harvard.edu (M.M.J.); siam.oottamasathien@childrens.harvard.edu (S.O.); 4 Department of Pathology, University of Utah, Salt Lake City, UT 84112, USA; Jolanta.Jedrzkiewicz@hsc.utah.edu; 5 Department of Pharmaceutics and Pharmaceutical Chemistry, University of Utah, Salt Lake City, UT 84112, USA; Joseph.Cappello@genelux.com; 6 Department of Urology, Massachusetts General Hospital, Harvard Medical School, Boston, MA 02114, USA

**Keywords:** radiation induced proctitis, semi-synthetic glycosaminoglycan ethers, silk-elastinlike polymers

## Abstract

Semisynthetic glycosaminoglycan ethers (SAGEs) are short, sulfated hyaluronans which combine the natural properties of hyaluronan with chemical sulfation. In a murine model, SAGEs provide protection against radiation induced proctitis (RIP), a side effect of lower abdominal radiotherapy for cancer. The anti-inflammatory effects of SAGE have been studied in inflammatory diseases at mucosal barrier sites; however, few mechanisms have been uncovered necessitating high throughput methods. SAGEs were combined with silk-elastinlike polymers (SELPs) to enhance rectal accumulation in mice. After high radiation exposure to the lower abdominal area, mice were followed for 3 days or until they met humane endpoints, before evaluation of behavioral pain responses and histological assessment of rectal inflammation. RNA sequencing was conducted on tissues from the 3-day cohort to determine molecular mechanisms of SAGE–SELP. After 3 days, mice receiving the SAGE–SELP combination yielded significantly lowered pain responses and amelioration of radiation-induced rectal inflammation. Mice receiving the drug–polymer combination survived 60% longer than other irradiated mice, with a fraction exhibiting long term survival. Sequencing reveals varied regulation of toll like receptors, antioxidant activities, T-cell signaling, and pathways associated with pain. This investigation elucidates several molecular mechanisms of SAGEs and exhibits promising measures for prevention of RIP.

## 1. Introduction

Radiation induced proctitis (RIP) is a common side effect of pelvic radiotherapies which aim to treat lower abdominal cancers such as prostate, uterine, vaginal, and cervical cancers [1]. The anatomical fixed position makes the rectum especially susceptible to exposure from ionizing radiation, commonly resulting in inflammation. Of the patients receiving lower abdominal radiotherapy, it is estimated that 5–20% will develop some form of acute and/or chronic RIP [2]. Acute RIP occurs within days to months following irradiation and can result in common symptomology of abdominal pain/cramps, diarrhea, hematochezia, and other adverse effects [2,3]. The occurrence of acute RIP can lead to cessation of radiotherapy schedules which are needed for cancer treatment. Chronic RIP typically develops months to years following irradiation, and is met with more debilitating symptoms of acute RIP along with possibilities of fistulas, incontinence, strictures, and adverse effects [2]. The full pathological development of RIP is still being elucidated. Upon irradiation, double stranded DNA breaks and cell membrane damage could lead to cell and tissue injury. Radiolysis of intracellular water leads to free radical generation and further tissue damages [4,5]. After just two weeks of initiating radiotherapy, histological changes have been observed in the clinic. These changes include inflammatory infiltrates, migration of nuclei, atypical mitoses, loss of intestinal stem cells, loss of glands, etc. [3]. Presentation of acute RIP increases the risk of late developing chronic proctitis by more than five-fold in patients following external beam radiotherapy for prostate carcinoma [6]. The resolution of acute RIP can result in characteristics of chronic proctopathy which include fibrosis and epithelial atrophy. Currently, there are limited therapeutic prophylactics which aim to modulate the presentation of acute inflammation found within RIP.

Semi-synthetic glycosaminoglycans are derived from naturally occurring glycosaminoglycans with additional molecular programming provided by synthetic modifications. Chemically sulfated hyaluronic acid-based molecules are one such example. Some of the programmed properties of sulfated hyaluronans include inhibition of hyaluronidase [7], blocking of P- and L-selectin [8], and increased interactions with BMP-4 and TGF-β1 [9,10]. Semi-synthetic glycosaminoglycan ethers (SAGEs), consisting of oligomeric sulfated hyaluronans, exhibit a variety of therapeutic mechanisms in dampening inflammatory injuries. Some of these mechanisms include blocking of pathogen associated molecular patterns (PAMPs) [11], release of extracellular ATP from urothelial cells [12], inhibition of the receptor for advanced glycation end products (RAGE) [13], and reduced inflammatory infiltrates into inflamed sinonasal epithelium [14]. A particular SAGE, GM-0111, has exhibited anti-inflammatory properties in mucosal settings such as interstitial cystitis [12,15], periodontitis [11], and RIP [16,17]. In a murine RIP model, rectal administration and accumulation resulted in a radioprotective effect against RIP, illustrated by decreased histological injury and symptomology as assessed via behavioral pain responses. This protection was made possible by delivery via a liquid to semi solid enema system, mediated by delivery via hydrogels made of silk-elastinlike protein polymers (SELPs) [16,17].

SELPs have been utilized to enhance the rectal bioaccumulation and efficacy of GM-0111 in a prophylactic murine RIP model [16,17]. SELPs are composed of motifs derived from *Bombyx mori* silk (GAGAGS) and human tropoelastin (GVGVP). The combination of these silk and elastin motifs provides SELPs with the ability for in situ crosslinking and thermoresponsive behavior [18]. Upon heating to body temperature, and depending on structure and concentration, SELPs undergo a rapid sol to gel transition, resulting in a robust crosslinked hydrogel. These crosslinks, mediated by silk motifs, are composed of antiparallel beta sheets held together through hydrophobic interactions and hydrogen bonding [19,20]. This passive crosslinking mechanism provides SELPs with injectability and biocompatibility, capable for use in local gene delivery [21,22], drug delivery [23], embolic applications [24], and cellular scaffolds [25]. Several analogs of SELPs have been produced with variation in silk and elastin sequences, providing opportunity to tune SELP gel mechanical properties, matrix characteristics, and control over spatiotemporal release kinetics in vivo [21]. In the context of RIP, SELP-415K (four silk units, 15 elastin units with one lysine substituted elastin unit per repeat) enhanced rectal accumulation of GM-0111 compared to phosphate buffered saline, Poloxamer 407, and another SELP analogue. This enhanced accumulation was attributed to sustained release over 12 h and slower gelation compared to other investigated polymers. In vivo this translates to GM-0111 accumulation through several mucus turnovers and a larger gel-tissue interface, respectively [16]. When mice were exposed to high doses of irradiation, this drug–polymer composition provided rectal protection after 7 days by limiting rectal injury, decreasing behavioral pain responses, and reducing radiotoxicities [16]. The aim of this study is to expand upon the protection provided by matrix-mediated delivery of GM-0111 via SELP-415K hydrogels. Due to the enhanced delivery provided by SELPs, we hypothesize that delivered GM-0111 will: (1) provide early protection against RIP, (2) sustain animal health, and (3) modulate the early pathological development of RIP.

## 2. Materials and Methods

### 2.1. Materials

SELP-415K was synthesized and processed as previously described [26,27,28,29]. GM-0111 was purchased from GlycoMira, Inc. (Salt Lake City, UT, USA). Phosphate buffered saline (PBS) was purchased from Sigma Aldrich (St. Louis, MO, USA). Female (8-week old) BDF-1 mice (stock no: 100006) were obtained from The Jackson Laboratory (Bar Harbor, ME, USA). All animal care and procedures were conducted in accordance with The University of Utah Institutional Animal Care and Use Committee policies [approval number: 20-03015 (2020)]. 

### 2.2. Mouse Treatment and Irradiation

Mice were treated as previously described [17]. Prior to procedures, mice were weighed and fasted overnight by removing all food and bedding from cages. The next morning mice were assigned randomly to experimental groups (*n* = 6). These included PBS, GM-0111 (100 mg/mL) in PBS (GM–PBS), SELP-415K 11 wt. %, and GM-0111 (100 mg/mL) in SELP-415K 11 wt. % (GM–SELP). Mice were anesthetized with 2% isoflurane. A catheter was inserted into rectums and up to 100 µL of each formulation was slowly instilled into the rectum of each mouse as the catheter was slowly retrieved from the cavity. Mice were then immobilized on an irradiation platform in a supine position. A lead plate was placed above the mice with 4 × 1 cm apertures to restrict irradiation to the lower abdominal region over the rectum. The anus of each mouse was aligned to the bottom of the aperture or immediately below it. Mice then received 37 Gy of irradiation using a RS 2000 X-ray irradiator (RAD SOURCE Technologies, Buford, GA, USA). Immediately following, mice were monitored for a recovery period before being placed back into cages with food and bedding. Mice were then monitored for signs of distress and loss of body weight. Mice were sacrificed at a 3-day timepoint or after loss of >20% of body weight. Animals not exhibiting excess body weight loss were sacrificed at a 14-day endpoint.

### 2.3. Behavorial Pain Testing

Before overnight fasting, mice were assessed for behavioral pain responses to obtain baseline response rates. Mice were placed in a mesh enclosure for at least 10 min prior to stimulation of the suprapubic area with 0.04, 0.16, 0.40, 1.0, and 4.0 g Von Frey filaments, as previously described [30]. To avoid wind up effects, each stimulation trial occurred at least 10 s apart. A total of 10 replicates for each filament strength were recorded. Positive responses included sharp abdominal retractions, jumping, vocalization, and/or immediate scratching/licking of the stimulated area. Behavioral pain responses were also evaluated immediately prior to sacrifice to assess the sensitivity of each mouse to mechanical stimulation.

### 2.4. Histology

The tissue sampling was performed on deceased animals 3 days after irradiation and at the survival endpoint determined by animal weight loss of >20% after irradiation (variable survival length). Rectal tissues were dissected grossly during necropsy and subsequently fixed in 10% neutral buffered formalin, before soaking in 70% ethanol for at least 24 h. Specimens were then submitted to Associated Regional and University Pathologists, Inc. (Salt Lake City, UT, USA) for sectioning, processing, and embedding into the paraffin blocks. Then, 2-µm sections were obtained and placed onto glass slides and stained with hematoxylin and eosin (H&E). 

All slides were evaluated histologically according to the previously described scoring method [3,31]. Evaluation included assessment of surface epithelium such as loss of cellular height and flattening of cells and cellular inflammatory infiltrates (in the form of neutrophils). Glandular composition was also assessed particularly the luminal migration of epithelial nuclei, loss of goblet cells, mitotic activity, cryptitis, eosinophilic crypt abscesses, loss of glands, atrophy of glands, and gland distortion. The lamina propria assessment included evaluation of inflammatory infiltrates, edema, and congestion of vasculature. The mitotic activity was scored as normal, increased, or decreased. The eosinophilic crypt abscesses, gland atrophy, and distortion were marked as absent or present. The remaining histologic abnormalities were scored on a scale of 0 to 4 (0 = normal, 1 = mildly abnormal, 2 = moderately abnormal, 3 = markedly abnormal, and 4 = severely abnormal). Additionally, an overall microscopic damage score was assigned based on histologic alterations identified with low-power magnification (mostly based on architectural abnormalities of glands and inflammatory infiltrates) and scored on a scale of 0 to 4 (0 = normal, 1 = mildly abnormal, 2 = moderately abnormal, 3 = markedly abnormal, and 4 = severely abnormal). Histologic assessment was performed in a blinded manner, as the pathologist was not aware of the treatment groups at the time of assessment [3,31]. Data was tabulated in Microsoft Excel spreadsheets (2111, Arlington, VA, USA).

### 2.5. RNA Sequencing

Upon animal sacrifice, at least 10 mg of rectal tissue was collected and flash frozen. Samples were then sent to GENEWIZ (South Plainfield, NJ, USA) for processing. Counts were collected with an Illumina HiSeq (Illumina, San Diego, CA, USA), 2 × 150 bp configuration, single index, per lane sequence configuration. Received counts were then processed as follows. A mouse genome (GRCm38) and gene feature files were obtained from the Ensembl release 102 [32]. A reference database was generated using STAR (2.7.6a, Cold Spring Harbor, NY, USA) with optimized splice junctions of 150 base pair reads. Reads were trimmed using cutadapt (1.16, Dortmund, Germany) [33] and aligned using STAR in two pass mode to the reference database. Reads were assigned to genes using featureCounts (1.6.3, Parkville, Australia) [34]. Output files were summarized with MultiQC (1.11 Stockholm, Sweden)to assess for outliers [35]. Differentially expressed genes were identified using a false discovery rate of 5% with DESeq2 (1.30.1, Heidelberg, Germany) [36] and pathways analyzed with Ingenuity Pathway Analysis (70750971, Qiagen, Hilden, Germany) [37]. Gene Ontology was assessed using EnrichR (March 2021 Version, New York, NY, USA) [38,39,40].

### 2.6. Statistical Analysis 

Behavioral response rates were analyzed with a 2-way ANOVA with a Bonferroni post hoc test. Nonparametric histological data was assessed using a Kruskal–Wallis test with a Dunn’s Multiple Comparison Test for group-to-group comparison. Survival trends were analyzed using a Mantel–Cox test and corrected for multiple comparisons [41,42]. Data is represented as mean ± standard deviation. Gene ontology *p*-values were computed using the Fisher exact test, assuming binomial distribution and independence for probability of any gene belonging to any set. Data was organized using Microsoft Excel, graphs prepared in GraphPad Prism (5.01, GraphPad, San Diego, CA, USA), and figures adapted for publication in Adobe Illustrator (23.0.1Adobe, San Jose, CA, USA). 

## 3. Results

### 3.1. Three-Day Behavorial Pain Responses

**Figure 1:**
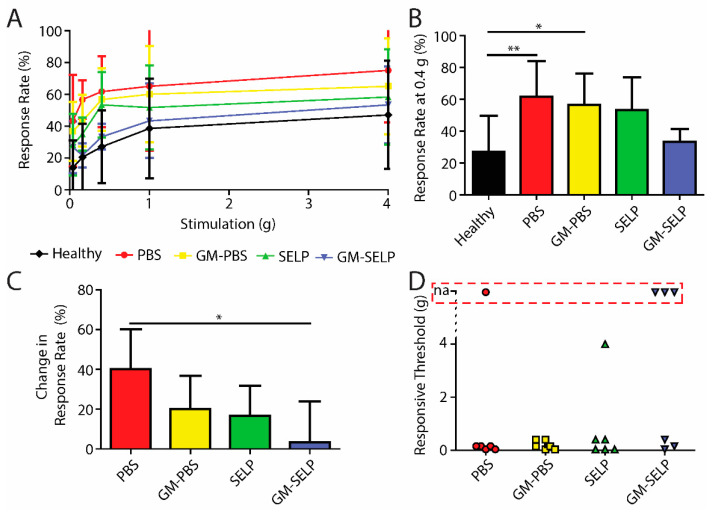
Behavioral pain responses 3-day post irradiation. (**A**) Response rates of irradiated animals receiving selected treatments and healthy controls. (**B**) Response rates with the 0.4-g filament. (**C**) Change in response rates from baseline measurements with the 0.16-g filament. (**D**) Threshold required to elicit an allodynic response as measured by an increase in 30% from the baseline. Red box indicates animals with no allodynic response (na) (** *p* < 0.01, * *p* < 0.05).

To evaluate the development of nociceptive response during the acute phase of RIP development, we assessed mechanical sensitivity 3 days after irradiation. All irradiated animals had signs of increased pain as assessed with Von Frey filaments (Figure 1A). At a stimulus of 0.4 g, the animals having received the PBS and GM–PBS compositions yielded significantly increased positive response rates (61.7 ± 22.3 and 56.7 ± 19.7%) compared to healthy control animals (27.1 ± 22.8%). At this stimulus force of 0.4 g, the animals receiving the GM–SELP composition only had a response rate of 33.3 ± 8.2% (Figure 1B). At 0.16-g stimulus, animals receiving GM–SELP (+3.3 ± 20.7%) had a significantly lower change in response rate compared to animals receiving PBS (+40.0 ± 20.0%). Groups receiving GM–PBS (+20.0 ± 16.7%) and SELP (+16.7 ± 15.1%) had insignificant changes to their sensitivity (Figure 1C). Analysis in this manner emphasizes the individual animal basis, as it normalizes to using baseline responses prior to treatment and irradiation. Interestingly, SELP alone exhibited some effect in reduction of behavioral pain responses. This has been observed in prior studies and may arise from maintenance of the mucus layer [16,17]. The degree of allodynia, or painful response to a normally not painful stimulus, was determined by the lowest level of stimulus required to achieve a 30% increase in response rate from baseline. Allodynia was observed in 20 out of 24 irradiated animals. Of the mice receiving GM–SELP, three out of six did not experience allodynia within the constraints of the Von Frey filament testing (0.04–4.0 g). Out of six mice, a single mouse receiving PBS also did not experience allodynia (Figure 1D). Of those mice experiencing typically non-normal pain, all groups receiving either GM–PBS (0.2 ± 0.16 g, *n* = 6), SELP (0.82 ± 1.57 g, *n* = 6), or GM–SELP (0.2 ± 0.18 g, *n* = 3) required higher thresholds for painful responses than mice receiving PBS (0.11 ± 0.7 g, *n* = 5). These outcomes indicate there was a prophylactic effect of GM-0111 after 3 days, especially in the context of enhanced bioaccumulation provided by SELP. 

### 3.2. Three-Day Histological Outcomes

**Figure 2:**
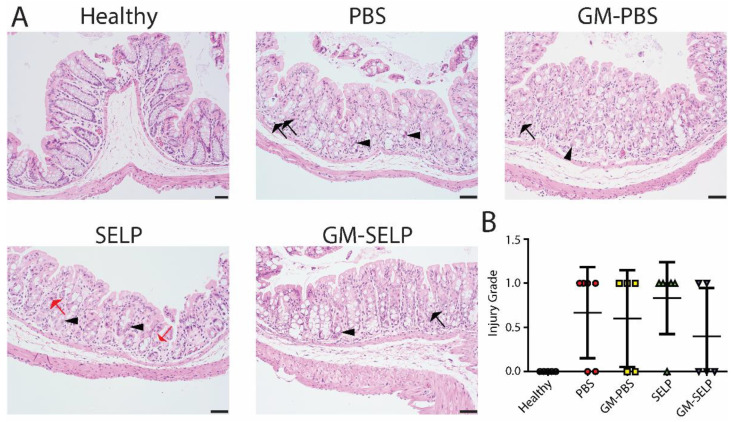
Histological analysis of tissues 3 days following irradiation. (**A**) Histological sections of healthy and irradiated animals receiving selected treatments. Black arrows indicate apoptosis, arrowheads indicate epithelial cell pleomorphism, and red arrows indicate mitoses. (**B**) Blinded scoring of histological sections to determine injury score.

The local early radioprotective effect provided by the GM–SELP combination was evaluated histologically 3 days after irradiation of rectal tissues in 24 animals. Microscopic assessment showed increased mitotic activity (*n* = 11), luminal migration of nuclei within epithelium (*n* = 14), and increased crypt apoptosis (*n* = 22) (Figure 2A). Additionally, there were variable inflammatory infiltrates within the lamina propria in a subset of samples (Supplemental Figure S1). All irradiated groups had increased injury scores compared to healthy controls (*n* = 5). The injury score of GM–SELP (0.4 ± 0.5) was reduced compared to all other irradiated animal groups (PBS: 0.7 ± 0.5, GM–PBS: 0.6 ± 0.5, SELP: 0.8 ± 0.4) when scored in a blinded manner (Figure 2B). The overall tissue damage identified histologically 3 days after irritation was mild and averaged 0.6 ± 0.5 (on 0–4 scale).

### 3.3. Animal Survival Curves

**Figure 3:**
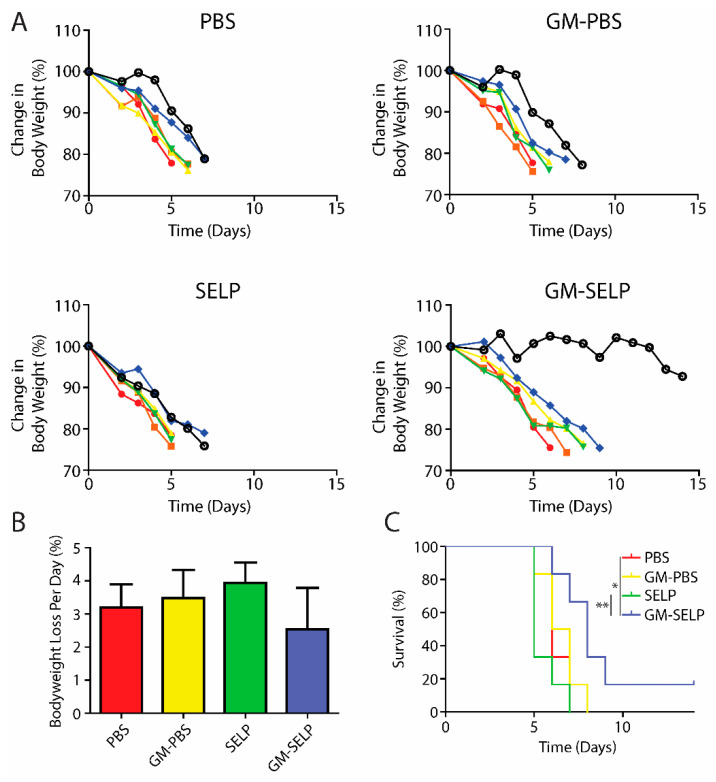
Animal body weights and survival. (**A**) Change in mice body weight as a percentage of baseline weights. Each line represents the body weight of a single animal. (**B**) Mean percentage of body weight loss per day. (**C**) Survival curves of irradiated animals receiving prophylactic treatments. (** *p* < 0.01, * *p* < 0.05).

To understand the protective benefits of SELP-mediated delivery at later time points, an additional cohort of animals was evaluated for long term survival following treatment and subsequent exposure to high doses of irradiation to the pelvic region. Animals were followed throughout the study for signs of radiotoxicities and declining health. The primary endpoint was >20% loss in body weight and animals were sacrificed if they crossed this threshold. The >20% weight loss was observed in nearly all irradiated animals, except for a single animal receiving the protective GM–SELP combination (Figure 3A). Over the study period, the GM–SELP combination resulted in the least amount of weight loss per day, compared to the other irradiated groups (PBS, GM–PBS, SELP) (Figure 3B), indicating a protective effect against radiotoxicities. Decreased weight loss per day resulted in an increased GM–SELP animal survival time. The median survival times of animals receiving PBS (6 days), GM–PBS (6.5 days), and SELP (5 days) were less than animals treated with GM–SELP, which survived 8 days (Figure 3C). GM–SELP improved mean survival time by 60%. A fraction of these animals (1/6) exhibited signs of long-term survival, maintaining body weight past the 14-day study period. The use of GM–SELP as a prophylactic resulted in significantly increased survival times when compared to SELP (*p* < 0.01) alone or the PBS (*p* < 0.05) sham treatment group (Figure 3C), suggesting a protective effect against early onset of RIP may be imperative for improved quality of life and delayed morbidity. 

### 3.4. Behavorial Pain Responses at Survival Endpoint

**Figure 4:**
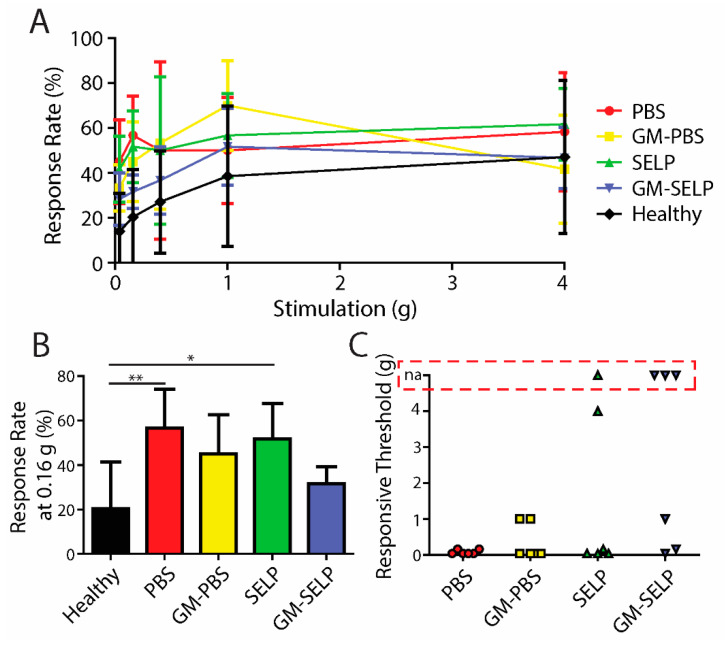
Behavioral pain responses at the time of sacrifice. (**A**) Response rates of irradiated animals receiving selected treatments and healthy controls. (**B**) Response rates with the 0.16-g filament. (**C**) Threshold required to elicit an allodynic response as measured by an increase in 30% from the baseline. Red box indicates animals with no allodynic response (na). (** *p* < 0.01, * *p* < 0.05).

Upon a >20% loss in body weight, mice in the survival group were tested for behavioral pain responses prior to sacrifice. At the time of sacrifice, response rates vary in the exact time following irradiation due to the nature of animal survival and defining a humane endpoint. Increases in response rates, for all irradiated animals, were observed compared to the healthy control group (Figure 4A). At a filament stiffness of 0.16 g, animals receiving GM–SELP (31.7 ± 7.5%) had the smallest increase in response rates compared to all other irradiated groups (Figure 4B). Animals receiving PBS (56.7 ± 17.5%) and SELP (51.7 ± 16.1%) had significantly higher response rates, at 0.16-g stimulus, than healthy animals (20.41 ± 21.03%) at the time of sacrifice (Figure 4B). Allodynia was also evaluated as described above (30% increase from baseline responses). Of these animals, 1/6 and 3/6 mice receiving SELP or GM–SELP did not exhibit allodynic responses, respectively. The remaining 20/24 animals exhibited allodynia as assessed with von Frey filaments. The stimulus threshold required to exhibit a 30% increase in response rate was much smaller in animals receiving PBS (0.08 ± 0.06 g, *n* = 6) compared to those animals receiving GM–PBS (0.36 ± 0.50 g, *n* = 6), SELP (0.86 ± 1.75 g, *n* = 5) or GM–SELP (0.40 ± 0.52 g, *n* = 3). This behavioral pain assessment at the time of sacrifice further illustrates the implications of the early stages of RIP in this murine model. The modulation of inflammation, pain pathways, or both before or at RIP onset has a sustained effect in this model as illustrated by reduced behavioral pain responses. 

### 3.5. Histology at Survival Endpoint

**Figure 5:**
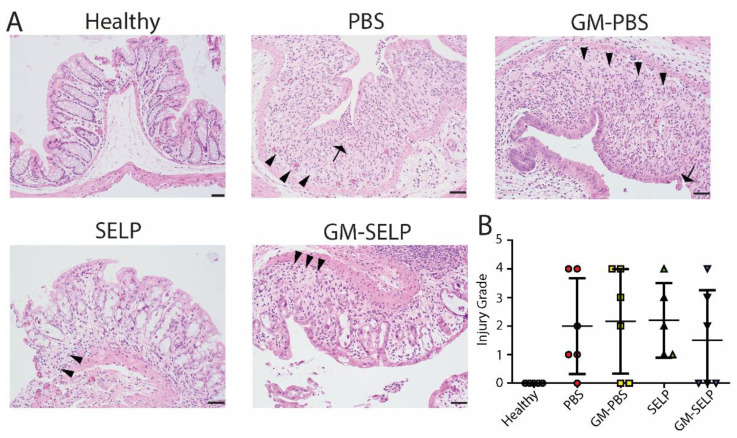
Time of sacrifice histological evaluation of rectal tissues. (**A**) Histological sections of healthy and irradiated animals receiving selected treatments. Arrows indicate epithelial erosion and arrowheads indicate crypt dropout. (**B**) Blinded scoring of histological sections to determine injury scores.

Histologic evaluation at the survival endpoint showed increased luminal migration of epithelial nuclei and variable inflammation in all irradiated groups. Additional alterations included cell flattening in the surface epithelium (*n* = 11), inflammatory infiltrates within the epithelium (*n* = 5), loss of goblet cells (*n* = 11), cryptitis (*n* = 6), loss of glands (*n* = 12), and edema (*n* = 9). Histologic alterations appeared more prominent at the survival endpoint as compared to the findings seen in samples evaluated three days after irradiation (Supplementary Figures S1 and S2) as illustrated by loss of crypts (crypt drop out) and epithelial erosion(s) (Figure 5A). The injury score of GM–SELP (1.5 ± 1.7) was reduced compared to all other irradiated animal groups (PBS: 2.0 ± 1.7, GM–PBS: 2.2 ± 1.8, SELP: 2.2 ± 1.3) when scored in a blinded manner (Figure 5B).

### 3.6. RNA Sequencing of Rectal Tissues

Tissue samples from irradiated mice receiving either PBS or the GM–SELP combination were collected at the 3-day time point and evaluated for gene expression using RNA sequencing. Differential gene expression and pathway analysis were conducted to understand the therapeutic mechanisms of GM-0111 in the protective approach to our RIP model. The use of the GM–SELP prophylactic in this RIP model resulted in 263 differentially expressed genes (adjusted *p* < 0.1) compared to mice receiving only PBS. Of these differentially expressed genes, 10 genes had a two-fold change greater than 1, and 67 had a two-fold change less than –1 (Supplementary Table S1). Indications of dampened immune activation are evident by decreased expression of chemoattractants, cytokines, interleukins, and associated receptors (CD83, IL27RA, IL9R, CD6, CCR7, IL2RG, CXCL13, CD52, CD4, CD84, IL16, etc.) (Supplementary Table S1). Pathway analysis of GM–SELP protection in mice revealed 91 significantly (−log(*p*) ≥ 1.3) enriched canonical pathways (Supplementary Table S2). Of these enriched pathways a total of five activated (Z-score ≥ 2) and six deactivated (Z-score ≤ −2) pathways were identified. The top 10 pathways with the highest absolute Z score revealed pathways involved with T lymphocytes, immune-based modulation, stress, and inflammation (Table 1). Immune pathways of T cell signaling (T-cell Receptor, PKCΘ, ICOS, and Th1) were deactivated compared to mice only receiving PBS. Increased antioxidant activities similar to Vitamin C are predicted, possibly owing to the polysulfated nature of GM-0111. Pathways specific to non-rectal tissues were excluded from this table. These and all other identified pathways can be found in Supplementary Table S2. These activated or deactivated pathways reflect potential therapeutic mechanisms and pathological results of the protective GM–SELP strategy. 

The differentially expressed genes were further used to identify key upstream regulators of the protective benefits of GM-0111 delivery via SELP. A total of 697 potential upstream regulators were identified to be significant (*p*-value of overlap <0.05) (Supplementary Table S3). Of these significant upstream regulators, 46 contained absolute Z-scores greater than 2 indicating differential activation status from mice receiving PBS only. Specifically, there were 33 deactivated (Z-score ≤ −2) and 13 activated upstream regulators (Z-score ≥ 2) when compared to mice only receiving PBS prior to irradiation. The top 10 most activated/deactivated upstream regulators, as determined by Z-score, include those associated with tumor necrosis factor, interferon gamma, aryl hydrocarbon receptors, lipopolysaccharides, colony stimulating factor 2, interleukin 10 receptor alpha (IL10RA), toll-like receptor 7 (TLR7), and CD28 (Table 2). Non-endogenous regulators were omitted from this table (Supplementary Table S3). Of these top 10 upstream regulators only IL10RA was activated, while all others were deactivated. Reduced involvement of pattern recognition receptors such as TLR-1,3,9,4 was predicted by IPA. Upstream immune regulators of both Th1 (TNF, IFNG, CSF2, and IL2) and Th2 (ILL6, IL1, and IL18) were all predicted to be deactivated (Z-score ≤ −2) via casual analysis (Supplementary Table S3). Together, these activation/deactivation statuses reflect the ameliorated immune response exhibited by delivered GM-0111. 

**Figure 6:**
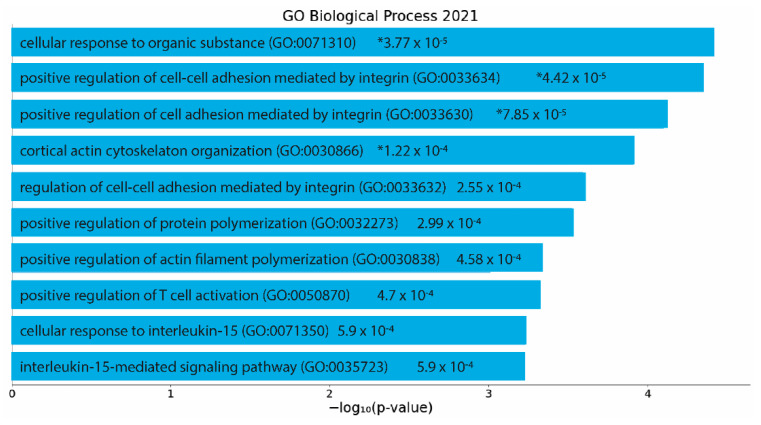
EnrichR analysis of differentially expressed genes (*p* adjusted < 0.05) with an absolute log2 fold change greater than 1. Gene ontology as determined via “GO Biological Process 2021”. *p*-values are listed on figure. *: adjusted *p* < 0.05.

To determine significantly enriched biological processes (*p* < 0.05), significant differentially expressed genes were then analyzed using EnrichR. Analysis of the protective action of the GM–SELP prophylactic revealed 185 significant biological processes (Supplementary Table S4). The top 10 gene ontology terms included regulation of cell adhesion, cytoskeletal reorganization, actin filament polymerization, and several immune cell signaling pathways (Figure 6). Of these enriched pathways, upstream regulators, and biological processes, a notable number were related to immune responses, emphasizing the immunomodulation capacity of GM-0111.

## 4. Discussion

RIP is an inadequately addressed form of rectal injury, resulting from lower abdominal radiotherapy for cancer. Radiation leads to damage and inflammation in the rectal tissue leading to a plethora of symptomology and tissue alterations. Previously, a GM–SELP combination was utilized in a prophylactic approach to protect mice against RIP and was evaluated after 7 days [16]. This study aimed to expand the beneficial effects of the GM–SELP prophylactic by studying short (3-day) and long (survival) term efficacy. Once in the mucosa, SAGE exhibits its therapeutic capabilities [16]. The enhanced spatiotemporal delivery provided by SELP results in increased rectal accumulation and therapeutic efficacy compared to saline formulations. By treating or protecting against acute RIP, further chronic complications can potentially be avoided.

In this study, histological changes were evident within three days of exposure to a high dose of irradiation. Pathologically, these changes were mild and include migration of apical nuclei, increased mitoses, and crypt apoptosis [3]. The observed nuclear migration and mitoses necessitate the use of cellular machinery such as actin filaments, a common gene ontological term identified by RNA sequencing. The degree of rectal injury was diminished when mice were protected with the SELP-mediated delivery of GM-0111. The histologic alterations appeared more pronounced at the time of sacrifice as compared to changes observed 3 days after irradiation, illustrating the need to define a specific timeframe for evaluation of RIP models. Modulation of the cytoskeleton through the Rho/Rho kinase pathway has been associated with fibrogenic smooth muscle cells in intestinal radiation damage [43]. This initial injury and dysregulation of the cytoskeleton precluded later pathological changes. In this instance at the time of sacrifice tissues exhibited a loss of goblet cells, cryptitis, eosinophilic crypt abscesses, inflammation, loss of glands, and edema (Supplemental Figure S2).

Upon irradiation, a plethora of changes lead to inflammatory events within the rectum. Damaged cells may release damage associated molecular signals. Pathogen associated molecular patterns may be presented due to loss of epithelial barrier and bacterial/viral invasion of native tissue. Previously, GM-0111 exhibited inhibition of TLR-2,4 [11]. Within this study, we have further identified TLR-1,3,9 as deactivated through casual analysis of differentially expressed genes, which can result in decreased transcription via IRF-7, IRF-3, and NF-KB. TLR-1,9 rely on a TLR adaptor, myeloid differentiation primary response 88, for downstream activation of transcription factors [44]. This adaptor is predicted to decrease activation with GM–SELP protection, as identified with casual analysis. Identification of upstream regulators also identifies liposaccharides as molecular mediators. These are common ligands for several TLRs. Mast cells have been implicated in the pathology of RIP [45]. IL-33, a potent mast cell activator, results in the upregulation of CCR7 [46]. Transcriptomics in this investigation identified CCR7 to have significantly decreased differential expression.

This investigation’s primary goal was to evaluate the short-term efficacy and potential molecular mechanisms behind the GM–SELP prophylactic effects for RIP. These mechanisms are largely due to the therapeutic activity of GM-0111 and are consistent with previously published studies regarding its anti-inflammatory properties. It is possible that SELP influenced the observed molecular mechanisms in this study as well. SELP hydrogels release soluble polymer fraction, however these amounts are lower compared to release of GM-0111 [21]. The pathology, as evaluated via histology, clearly develops beyond 3 days and up until sacrifice. Animals studied at the time of sacrifice provide an understanding of animal health when >20% loss of body weight was observed. The variation in sacrifice times makes it difficult to make direct comparisons and establish pathological development within a specific time frame. The rapid decline of animal health in this investigation illustrates the effects of extreme doses of radiation on mice and presenting effects in late responding tissues to ionizing radiation. Within the aperture and radiodose provided, it is likely that parts of the lower intestine were irradiated and damaged, although this was not directly observed. These non-rectal tissue effects make this model an unlikely candidate for evaluation of chronic RIP. Improvement of the current model could capitalize on animal positioning to limit intestinal exposure, optimization of total radiodose, and fractionation to limit slow developing tissue effects. Future studies will focus on: (1) validating the molecular mechanisms of GM–SELP discovered in this investigation by microarray and proteomic analyses, (2) establishing a model capable of evaluating outputs in the context of chronic RIP, (3) evaluating the efficacy of GM–SELP on treatment strategies following irradiation, (4) assessing the efficacy of GM–SELP in improving pathologies and symptoms from established chronic RIP, and (5) determining the pharmacokinetics and biodistribution of rectally delivered GM-0111. Pharmacokinetic and biodistribution studies will inform off target and systemic exposures of GM-0111 and possibly SELP-415K. Once determined further safety and toxicology investigations should be conducted depending on the biodistribution of GM-0111. These could include pulmonary, cardiovascular, renal, hepatic, and more toxicological assessments. If presented in the hepatic and renal regions, functionality tests should be assessed as well. While the GM–SELP combination does not directly interact with vasculature, it is likely that GM-0111 may enter the blood stream. To this end, hemocompatibility should be assessed at relevant concentrations that may be ascertained during pharmacokinetic and biodistribution studies. A low concentration of SELP may be taken up into systemic circulation. SELPs have been used prior for liquid embolic applications, and illustrate minimal activation of the complement system, hepatoxicity, and hemolysis [24,47]. Obtaining complete blood profiles in future works could provide another indicator of safety and radiotoxicities.

In the clinic, this SAGE–SELP combination can be used as a protective strategy administered several hours or immediately prior to radiotherapy. This can take the form of a coating agent applied directly to the rectal tissue or as a liquid to semi-solid enema. The protective success of this combination within the current model provides an indication for prophylactic intervention in patients undergoing hypo-fractionated radiotherapy. This is commonly used for cancers with a low α/β ratio, such as prostate cancers [48]. Additionally, the protection provided by GM–SELP may allow for dose escalation in other cancer types, further improving anti-tumor efficacies while minimizing rectal toxicities.

Pain is a common symptom of RIP and clearly develops with the model utilized in this study. The exact drivers of this pain are not known but may be associated with inflammation, pain pathways, or both. A possible explanation of the observed decrease in pain could be attributed to a reduced injury score of the rectal tissue. However, enriched canonical pathways also suggest other mechanisms acting in parallel or downstream of inflammation. The Insulin Receptor Signaling Pathway has also been implicated in neuropathic pain as evidenced in numerous studies of diabetic neuropathy [49]. Vitamin C has exhibited analgesic properties in the clinic, which is hypothesized to result from its antioxidant properties and subsequent scavenging of free radicals [50]. Similar antioxidant properties and scavenging of free radicals may be presented from GM-0111 owing to its sulfate group. This is especially pertinent in the setting of RIP due to the reactive species generated during radiolysis and inflammation. The Corticotropin Releasing Hormone Pathway, identified in this analysis, is also implicated in nociceptive pain. Upon activation in the hypothalamus, this pathway results in secretion of corticosteroids to peripheral sites for analgesic action [51]. Interestingly, analyses indicate an activated state of this pathway, raising the question of its involvement in RIP and the role GM-0111 plays in activating these potential analgesic capabilities.

## 5. Conclusions

A drug–polymer combination (GM–SELP) can provide acute protection against pain and rectal inflammation in a RIP model utilizing high doses of radiation. Prophylactic protection with GM–SELP translated into a 60% prolonged survival. Animals receiving the drug–polymer combination exhibited decreased pain and rectal inflammation. Assessment of the molecular basis of this protection includes, but not limited to, decreased activation of inflammatory pathways associated with pattern recognition receptors, lymphocyte signaling, antioxidant properties, and effects of lipopolysaccharides.

## Figures and Tables

**Table 1:** Canonical pathways identified from protection of GM–SELP in a RIP model.

Ingenuity Canonical Pathways	−log (*p*-Value)	Ratio	Zz-Score	Molecules
PKCθ Signaling in T Lymphocytes	1.65	0.0179	−2.646	CACNA1I, CACNG1, CARD11, CD4, HLA-A, HLA-DMB, HLA-DRA, HLA-DRB5, NFKBIA, Trbc1
Crosstalk between Dendritic Cells and Natural Killer Cells	5.94	0.0879	−2.449	CCR7, CD83, CSF2RB, HLA-A, HLA-DRA, HLA-DRB5, IL2RG, ITGAL
Th1 Pathway	3.19	0.0492	−2.449	CD4, HLA-A, HLA-DMB, HLA-DRA, HLA-DRB5, IL27RA
PD-1, PD-L1 cancer immunotherapy pathway	2.66	0.0472	2.236	HLA-A, HLA-DMB, HLA-DRA, HLA-DRB5, IL2RG
Senescence Pathway	1.37	0.0202	2.236	CACNG1, CAPN9, HBP1, PDHA1, PDHB, PPP2R5A
Corticotropin Releasing Hormone Signaling	2.03	0.0336	2	ADCY9, CACNA1I, CACNG1, SLC39A7, SMO
Antioxidant Action of Vitamin C	1.82	0.036	2	CSF2RB, NFKBIA, PLA2G2D, PLCB2
Insulin Receptor Signaling	1.49	0.0286	2	GAB1, PPP1CB, RHOQ, SHC1
T Cell Receptor Signaling	2.6	0.0212	−1.941	CARD11, CD4, CD8B, HLA-A, HLA-DMB, HLA-DRA, HLA-DRB5, ITGAL, NFKBIA, PTPN6, PTPN7, SKAP1, Trbc1
ICOS-ICOSL Signaling in T Helper Cells	1.51	0.0178	−1.89	CD4, HLA-A, HLA-DMB, HLA-DRA, HLA-DRB5, IL2RG, NFKBIA, SHC1, Trbc1

**Table 2:** Upstream regulators identified from protection with GM–SELP in RIP model.

Upstream Regulator	Activation Z-Score	*p*-Value of Overlap	Molecules
TNF	−3.784	0.00334	ACADM, BCL2A1, BTBD3, CAMP, CCL5, CCR7, CD4, CD83, CDK5R1, CSF1R, CSF2RB, CX3CR1, CXCL13, CXCL2, GAB1, Glycam1, HLA-A, HLA-DRA, IL16, IL27RA, ITGAL, KCNJ2, LAMP3, MUC2, NFKBIA, PLK3, SMO, VCL, ZNF750
IFNG	−3.694	0.000314	ABCB1, BCL2A1, C1QB, CCL5, CCR7, CD4, CD83, CDK5R1, CSF1R, CSF2RB, CX3CR1, CXCL2, HCK, HLA-A, HLA-DMB, HLA-DRA, HLA-DRB5, Ighg3, ITGAL, Klrk1, LAMP3, LAT2, MUC2, NFKBIA, PDHA1, PTPN6, RAB27A
AHR	−3.268	0.000668	C1QB, CARD11, CCL5, CCR7, CD4, CD8B, CSF2RB, CX3CR1, CXCL13, CXCL2, IL9R, VCL
Lipopolysaccharide	−3.193	0.000000174	ABCB1, ACADM, BCL2A1, CAMP, CCL5, CCR7, CD4, Cd52, CD83, CDK5R1, CNNM2, CNST, CSF1R, CSF2RB, CX3CR1, CXCL13, CXCL2, FBN1, GAB1, GIMAP7, HACD2, HCK, HLA-A, HLA-DMB, HLA-DRB5, IER5, Ighg3, IKZF1, IL16, IL2RG, ITGAL, ITPKC, LAMP3, LYZ, MUC2, NFKBIA, PLA2G2D, PLK3, PPP1CB, PTPN7, TFDP2, TNFRSF13B, VCL
IL6	−3.064	0.000261	BTC, CCL5, CCR7, CD83, CSF1R, CSF2RB, CX3CR1, CXCL13, CXCL2, HLA-A, HLA-DRB5, IL2RG, LYZ, NFKBIA, PLA2G2D, RAB27A, RNASE6, SMO
*E. coli* B4 lipopolysaccharide	−2.918	0.00022	C1QB, CCL5, CCR7, Cd52, CD83, CXCL2, HLA-A, LCP1, NFKBIA, PTPN6
CSF2	−2.905	0.00291	BCL2A1, CARD11, CCR7, CD83, CSF1R, CSF2RB, CX3CR1, CXCL2, IL16, LAMP3, LCP1, NFKBIA
IL10RA	2.813	0.00667	ABCB1, CCL5, CCR7, FBN1, HLA-A, IL2RG, Klrk1, Treml4
TLR7	−2.764	0.00014	ACAP1, BCL2A1, CCL5, CCR7, CD83, CXCL13, CXCL2, NFKBIA, PTPN6
CD28	−2.621	0.0385	BCL2A1, CXCL13, CXCL2, IL27RA, ITGAL, LCP1, NFKBIA

## Data Availability

Gene expression can be found within the NCBI BioSample database biorepository (Submission ID: SUB10548680, BioProject ID: PRJNA786943). BioSample accessions IDS and corresponding URLS are: 23763099: https://www.ncbi.nlm.nih.gov/sra/23763099; 23763100: https://www.ncbi.nlm.nih.gov/sra/23763100; 23763101: https://www.ncbi.nlm.nih.gov/sra/23763101; 23763102: https://www.ncbi.nlm.nih.gov/sra/23763102; 23763103: https://www.ncbi.nlm.nih.gov/sra/23763103; 23763104: https://www.ncbi.nlm.nih.gov/sra/23763104; 23763105: https://www.ncbi.nlm.nih.gov/sra/23763105; 23763106: https://www.ncbi.nlm.nih.gov/sra/23763106; 23763107: https://www.ncbi.nlm.nih.gov/sra/23763107; 23763108: https://www.ncbi.nlm.nih.gov/sra/23763108; 23763109: https://www.ncbi.nlm.nih.gov/sra/23763109; 23763110: https://www.ncbi.nlm.nih.gov/sra/23763110; 23763111: https://www.ncbi.nlm.nih.gov/sra/23763111; 23763112: https://www.ncbi.nlm.nih.gov/sra/23763112; 23763113: https://www.ncbi.nlm.nih.gov/sra/23763113; 23763114: https://www.ncbi.nlm.nih.gov/sra/23763114; 23763115: https://www.ncbi.nlm.nih.gov/sra/23763115; 23763116: https://www.ncbi.nlm.nih.gov/sra/23763116.

## Supplementary Materials

The following are available online at https://www.mdpi.com/article/10.3390/pharmaceutics14010175/s1, Figure S1: 3-Day Histological Scoring, Figure S2: Time of Sacrifice Histological Scoring, Table S1: Differentially Expressed Genes, Table S2: Canonical Pathways, Table S3: Upstream Regulators, Table S4: Gene Ontology Terms.
