# Supplementary material for: An Oligomeric Sulfated Hyaluronan and Silk-Elastinlike Polymer Combination Protects against Murine Radiation Induced Proctitis
Source: Pharmaceutics. 2022 Jan 12;14(1):175. doi: 10.3390/pharmaceutics14010175 (PMC8777937; doi:10.3390/pharmaceutics14010175)
Supplement: Supplementary file 1 [file pharmaceutics-14-00175-s001.zip › Supplementary Figures.pdf]

Supplementary Materials: An Oligomeric Sulfated Hyaluronan and Silk-Elastinlike Polymer Combination Protects against Murine Radiation Induced Proctitis

Douglas Steinhauff, Mark Martin Jensen , Ethan Griswold, Jolanta Jedrzkiewicz, Joseph Cappello, Siam Oottamasathien and Hamidreza Ghandehari

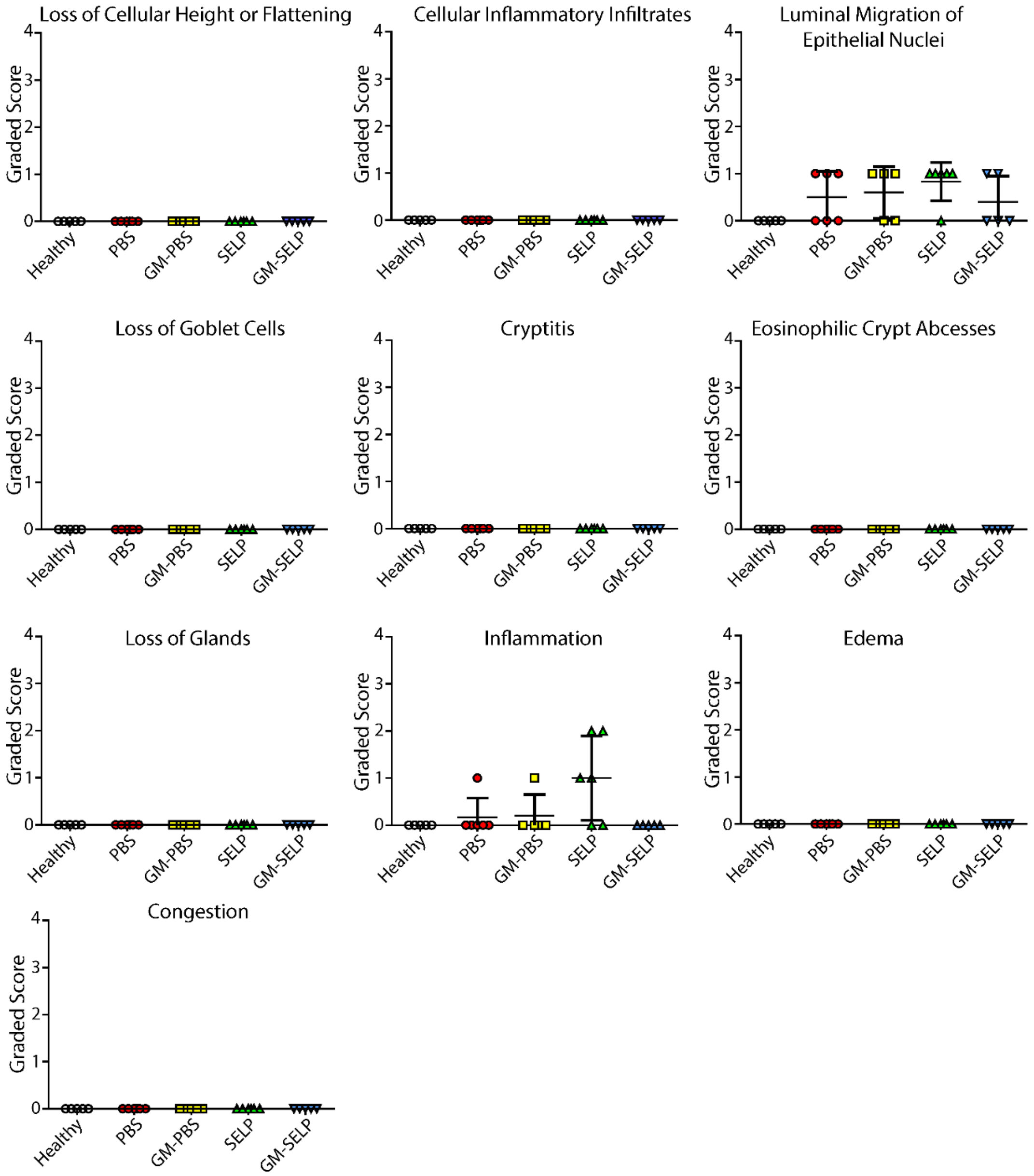

Supplementary Figure S1: Scoring of histological observations 3 days after treatment and irradiation. Scoring was performed in a blinded manner.

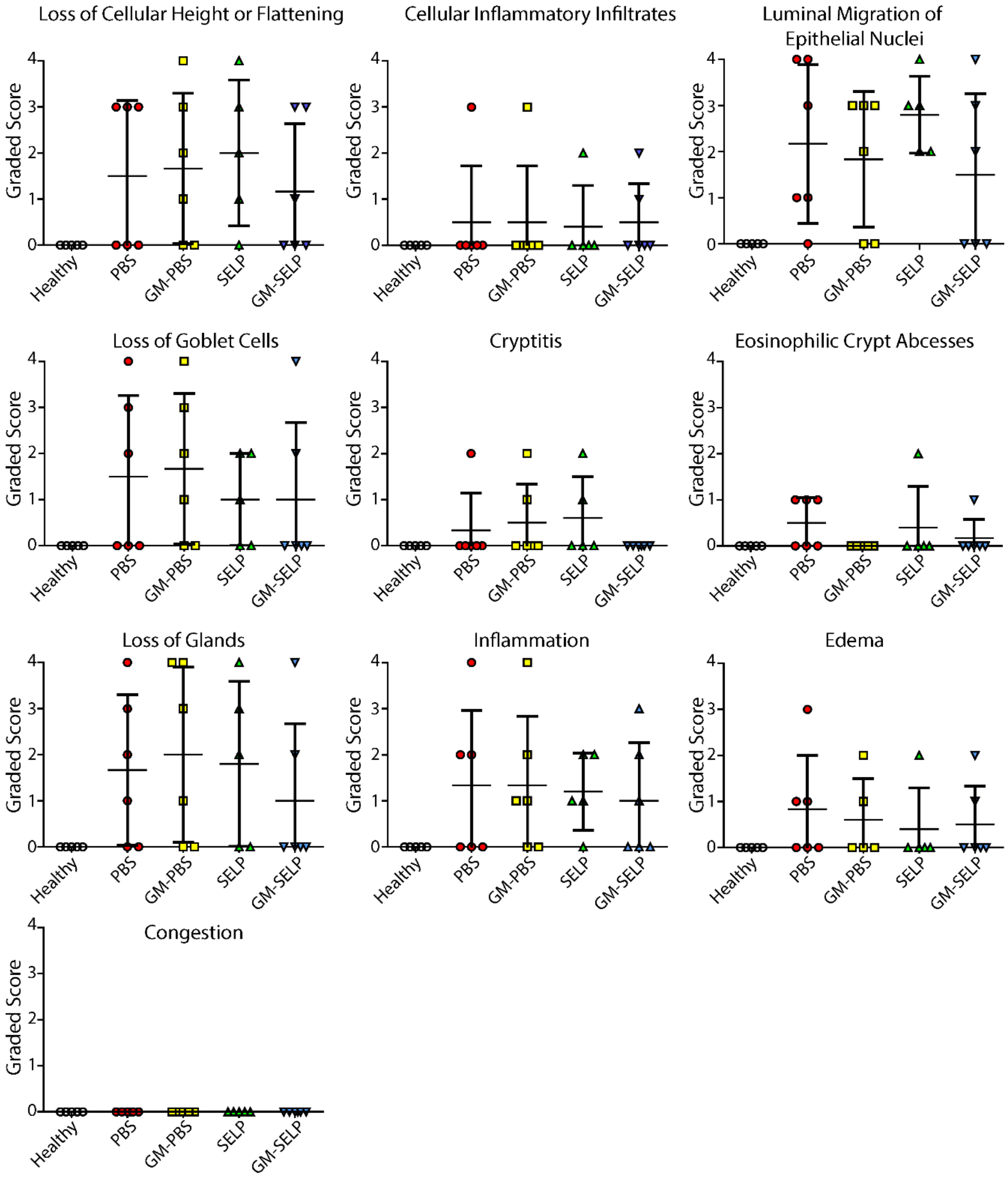

Supplementary Figure S2: Scoring of histological observations at the time of sacrifice for mice receiving treatment and irradiation. Scoring was performed in a blinded manner.
